# Effects of wearing a transparent face mask on perception of facial expressions

**DOI:** 10.1177/20416695221105910

**Published:** 2022-06-15

**Authors:** Yuki Miyazaki, Miki Kamatani, Tomokazu Suda, Kei Wakasugi, Kaori Matsunaga, Jun I. Kawahara

**Affiliations:** Department of Psychology, 12777 Fukuyama University, Fukuyama, Japan; Graduate School of Letters, 12810 Hokkaido University, Sapporo, Japan; Global Research & Development Division, Unicharm Corporation, Kanonji, Japan; Graduate School of Letters, 12810 Hokkaido University, Sapporo, Japan

**Keywords:** facial expression, face mask, transparent mask, COVID-19

## Abstract

Wearing face masks in public has become the norm in many countries post-2020. Although mask-wearing is effective in controlling infection, it has the negative side effect of occluding the mask wearer’s facial expressions. The purpose of this study was to investigate the effects of wearing transparent masks on the perception of facial expressions. Participants were required to categorize the perceived facial emotion of female (Experiment 1) and male (Experiment 2) faces with different facial expressions and to rate the perceived emotion intensity of the faces. Based on the group, the participants were assigned to, the faces were presented with a surgical mask, a transparent mask, or without a mask. The results showed that wearing a surgical mask impaired the performance of reading facial expressions, both with respect to recognition and perceived intensity of facial emotions. Specifically, the impairments were robustly observed in fear and happy faces for emotion recognition, and in happy faces for perceived intensity of emotion in Experiments 1 and 2. However, the impairments were moderated by wearing a transparent mask instead of a surgical mask. During the coronavirus disease 2019 (COVID-19) pandemic, the transparent mask can be used in a range of situations where face-to-face communication is important.

## Introduction

The World Health Organization has recommended wearing a face mask in public based on evidence that mask-wearing is effective in controlling the spread of coronavirus disease 2019 (COVID-19) (Howard et al., 2021; Rader et al., 2021; Ueki et al., 2020). Due to this, mask-wearing has become the norm in many countries post-2020 (Institute for Health Metrics and Evaluation, 2021). Given that nonpharmaceutical interventions, such as wearing a face mask, are needed at least one year after the start of vaccination (Yang et al., 2021), and vaccine effectiveness against new variants that may arise is unknown (Centers for Disease Control and Prevention, 2021), face masks will be required on a daily basis for some time.

While mask-wearing is effective in controlling infection, many researchers have pointed to its negative side effects in interpersonal situations due to the partial occlusion of critical parts of the face. The occlusion significantly impairs recognition of facial emotions (Carbon, 2020; Gori et al., 2021; Grenville & Dwyer, 2022; Grundmann et al., 2021; Kim et al., 2022; Marini et al., 2021; Noyes et al., 2021; Parada-Fernández et al., 2022; Pazhoohi et al., 2021; Ramachandra & Longacre, 2022) and perception of facial emotion intensity (Pazhoohi et al., 2021; Ramachandra & Longacre, 2022; Sheldon et al., 2021; Tsantani et al., 2022). These negative effects on emotional facial communication occur because the features of the bottom half of the faces that signal effective cues for reading others’ facial expressions (Blais et al., 2012; Bombari et al., 2013; Boucher & Ekman, 1975; Calder et al., 2000; Calvo et al., 2014; Eisenbarth & Alpers, 2011; Hanawalt, 1944; Kotsia et al., 2008; Saumure et al., 2018; Schurgin et al., 2014; Schyns et al., 2002; Wegrzyn et al., 2017) are occluded by the masks. The mouth region plays a critical role in expressing (and thus eventually perceiving) happy faces, while the contribution of the mouth region to expressing other emotional faces vary among previous studies (Boucher & Ekman, 1975; Calder et al., 2000; Calvo et al., 2014; Eisenbarth & Alpers, 2011; Hanawalt, 1944; Kotsia et al., 2008; Saumure et al., 2018; Schurgin et al., 2014). Consistent with these findings, previous studies have reported that the recognition of happy faces is impaired by mask-wearing (Carbon, 2020; Grenville & Dwyer, 2022; Kim et al., 2022; Marini et al., 2021; Noyes et al., 2021; Parada-Fernández et al., 2022; Pazhoohi et al., 2021; Ramachandra & Longacre, 2022).

Noteworthy, several studies investigating the effects of wearing a face mask on emotion recognition have been published after the onset of COVID-19 (Pavlova & Sokolov, 2022), and most of them were conducted in Western countries with one exception (Kim et al., 2022). These studies were performed using images of Caucasian faces and recruited Western participants. In fact, the issue is the same in the studies testing the emotion recognition by using the trimmed or occluded parts of the face (e.g., Calvo et al., 2014; Kotsia et al., 2008). The perception of facial expressions differs across Western and East Asian populations. This difference is based on the fact that East Asians and Westerners depend on different perceptual cues when reading facial expressions. Specifically, East Asians tend to focus on the eyes rather than on the mouth, while Westerners usually look at the whole face including the mouth (Jack et al., 2009; see also, Jack, 2013; Yuki et al., 2007). Considering East Asians’ dependency on cues around the eyes when reading emotions, they may be able to accurately recognize emotions even from masked faces. Therefore, it is important to test the external validity of previous studies by using East Asian faces and recruiting East Asian participants.

The negative effects of mask-wearing on emotion recognition have been known from the beginning of the spread of COVID-19, and several researchers have suggested copings such as utilizing body language (Mheidly et al., 2020). Body language is an effective way to convey emotions because it modulates perceived facial expressions (Aviezer et al., 2008). However, this coping does not provide a principled solution to the occlusion of facial expressions. People feel distressed not seeing each other's entire face or facial expressions (Molnar-Szakacs et al., 2021; Reidy et al., 2020; also see, PPE portrait project, 2021; Wiesmann et al., 2021). This lack of face-to-face contact is not solved by body language. One possible solution is to use a face shield instead of a face mask. However, while this may be effective in protecting the eyes (Chu et al., 2020), wearing a face shield without a face mask does not suppress respiratory droplets escaping from gaps (Verma et al., 2020). Therefore, wearing a face shield alone is ineffective in protecting against infection.

**Figure 1.**
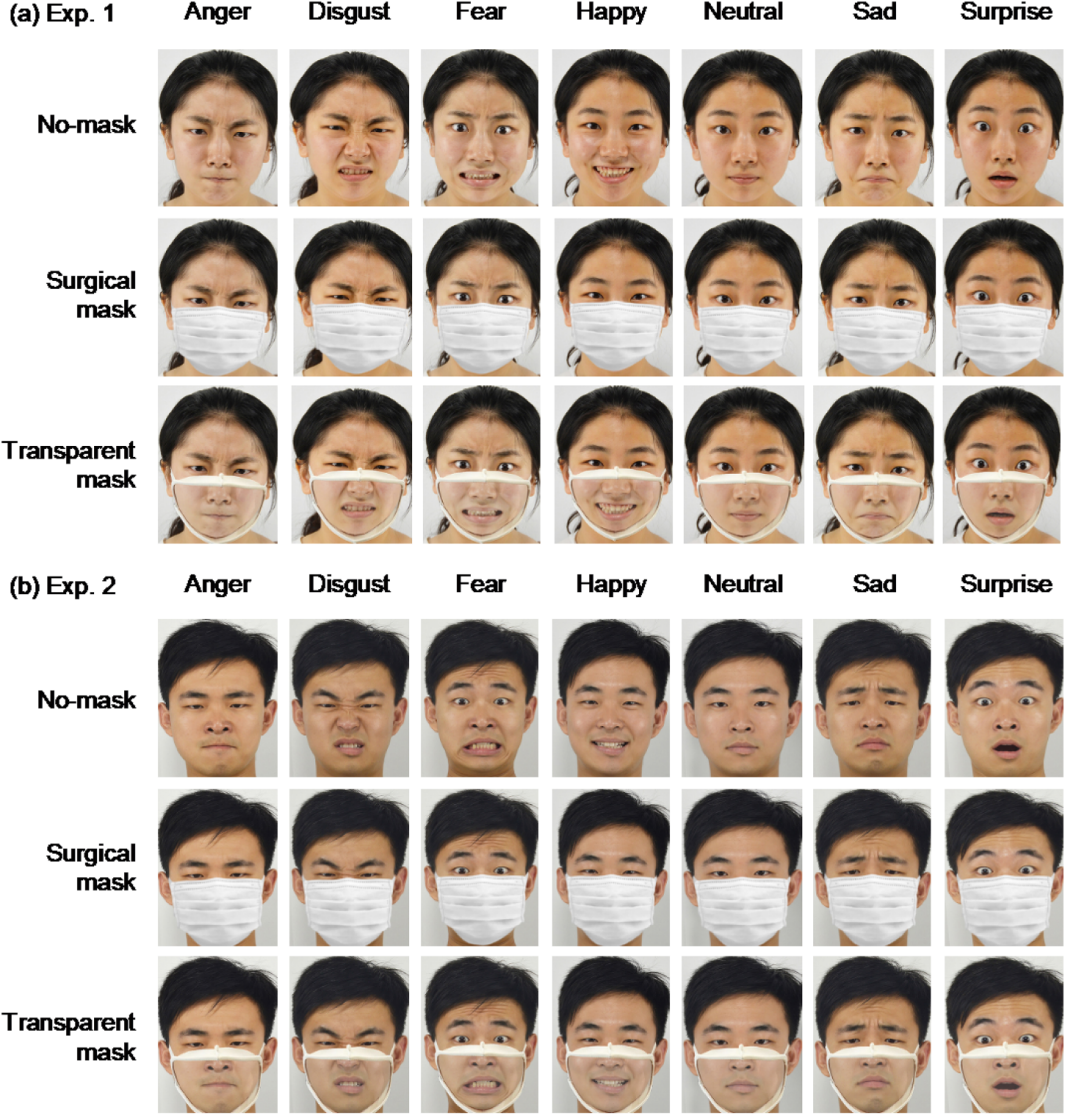
Examples of face stimuli used in Experiments 1 and 2 are shown in the top (a) and bottom (b), respectively. All stimuli can be seen on the OSF (https://osf.io/xsfg8/). Original images were drawn from Tsinghua facial expression database (Yang et al., 2020) with permission.

Here, we focus on transparent face masks (hereafter referred to as a transparent mask) as a solution to the occlusion of facial expressions. The transparent mask is a commercially available product in which the front is covered with a transparent film (e.g., plastic sheet, Figure 1, bottom row), allowing the lower half of the wearer's face to be clearly visible. To avoid gaps, the transparent mask was designed to fit tightly from the nose to the chin. Thus, the transparent mask is superior to face shields in preventing the splashing of respiratory droplets. There has been a dramatic increase in the demand for transparent masks since 2020, and the introduction of self-made transparent masks on social media sites (e.g., the DHH mask project, 2020). In the present study, we investigated whether a commercially available transparent mask could protect against the negative side effects of surgical masks on reading facial expressions. It is reasonable to assume that wearing a transparent mask (instead of a nontransparent one) makes it easier for other people to read facial emotions. However, it is also possible that wearing a transparent mask negatively affects emotion recognition (particularly, of happy faces) compared to not wearing a face mask. This is because wearing a transparent mask impairs the perceived attractiveness of faces (De Boeck & Vaes, 2021), which, in turn, moderates the processing of facial expressions (Golle et al., 2014; Taylor & Bryant, 2016). Specifically, the discrimination accuracy of unattractive happy faces is more decreased than that of attractive happy faces when choosing a happier face (Golle et al., 2014). Considering the influence of attractiveness on processing facial expressions, the emotion recognition of happy faces could be impaired depending on the reduction of attractiveness resultant from wearing a transparent mask. Consequently, it is necessary to compare the effect of wearing a transparent mask with that of not wearing a mask.

To summarize, the purpose of the current study was to investigate the effects of wearing a transparent mask on the perception of facial expressions. There were two main questions to be answered. We first tested whether recognition and perceived intensity of facial emotions when wearing a transparent mask were higher and comparable to when wearing a surgical mask and not wearing a mask, respectively. Second, we tested the replication probability of previous studies which stated that wearing a surgical mask impairs recognition and perceived intensity of facial emotions when compared to not wearing a mask. We investigated these questions in two online experiments. Experiment 1 was tested using female face stimuli, and the preregistered Experiment 2 was conducted using male face stimuli to examine whether the results in Experiment 1 could be replicated. Being able to see the mouth region is crucial to recognize a happy face (Boucher & Ekman, 1975; Calder et al., 2000; Calvo et al., 2014; Eisenbarth & Alpers, 2011; Hanawalt, 1944; Kotsia et al., 2008; Saumure et al., 2018; Schurgin et al., 2014). In addition, since many previous studies have reported impairments in emotion recognition (Carbon, 2020; Grenville & Dwyer, 2022; Kim et al., 2022; Marini et al., 2021; Noyes et al., 2021; Parada-Fernández et al., 2022; Pazhoohi et al., 2021; Ramachandra & Longacre, 2022) and perceived emotion intensity (Pazhoohi et al., 2021; Ramachandra & Longacre, 2022; Sheldon et al., 2021; Tsantani et al., 2022) of happy faces when wearing a surgical mask, we predicted to observe these impairments. We also expected that these impairments would be moderated by a transparent mask.

## Experiment 1

### Method

#### Participants

We used a two-way mixed analysis of variance (ANOVA; three face mask groups × seven facial expression conditions) and simple effect tests for each facial expression to examine the effect of the type of face mask on the perception of facial expressions. To detect the medium-sized effect of the type of face mask for each facial expression, we decided to recruit at least 159 participants (effect size *f* = 0.25, α = .05, 1 − β = .80, the number of groups = 3). The sample size was calculated using G*power 3 (Faul et al., 2007, 2009). To account for data exclusion (see below for the data acceptance criteria), we decided to recruit 300 volunteers via a Japanese crowdsourcing service. Finally, data from 261 online volunteers (151 females, 108 males, and 2 other sexes; *M*_age_ = 39.0 years and *SD*_age_ = 8.8 years) who passed the data acceptance criteria were used in the statistical analyses. This research project was approved by the research ethics committee of the last author's institution.

#### Design

The independent variables were the type of face mask (three levels: no-mask, surgical mask, and transparent mask groups) and the facial expression of the stimulus (seven levels: anger, disgust, fear, happy, neutral, sad, and surprise faces). The type of face mask was a between-participants factor, and the facial expression was a within-participants factor. Participants were randomly assigned to one of the three face mask groups (no-mask group, *n* = 94; surgical mask group, *n* = 83; transparent mask group, *n* = 84). The order of the seven facial expressions was randomized across the participants.

#### Face Stimuli

Face images of four East Asian young female models with seven facial expressions were selected from the Tsinghua facial expression database (Yang et al., 2020) with permission. The numbers of face models were Y23F, Y26F, Y48F, and Y52F. In the no-mask group, the 28 original images were used as the face stimuli. We digitally edited the face stimuli in both the surgical mask and transparent mask groups using a graphic editor (Adobe Photoshop 22.4, Adobe Inc.). The image used in the surgical mask group was a nonwoven surgical mask product (*Cho-kaiteki*® [ultra-comfortable] mask, pleated and regular type, Unicharm Corporation), and the image used in the transparent mask group was a transparent mask product (*Kao-ga-mie* [face-visible] mask, Unicharm Corporation). The images of the surgical and transparent masks were positioned in approximately the same area and shape within a face stimulus (Figure 1). In addition to these images, different face images with seven facial expressions using a different model (Y6F) were drawn from the same database for practice trials and an attention check trial. All face stimuli were downsized to 300 × 400 pixels. Figure 1(a) shows these face images. They are also shown on the OSF (https://osf.io/xsfg8/).

#### Procedure

The online experimental environment was created using the Qualtrics software. The participants first performed seven practice trials. The practice trials were the same among the three face mask groups. In each trial, a randomly selected face stimulus with one of the seven facial expressions was presented without a face mask. Participants were required to categorize perceived facial emotion of a stimulus from a list of seven emotions (anger, disgust, fear, happy, neutral, sad, and surprise), and rate the perceived intensity of emotion of the stimulus using a linear slider from 0 (*extremely weak*) to 100 (*extremely strong*) at their leisure. The face stimulus was presented one at a time on the screen, and the next face stimulus appeared after the responses. Participants were not told that these seven trials were for practice.

Participants performed 29 test trials (four face models, seven facial expressions, and one attention check) directly and continuously following the practice trials. Additional instructions, if any, were provided before initiating the test trials, depending on the face mask group. Participants assigned to the surgical mask group were instructed to respond in the same way as in the practice trials, even though the face stimuli would be presented with a surgical mask. Participants assigned to the transparent mask group were instructed to respond in the same way as in the practice trials, even though the face stimuli would be presented with a transparent mask. Example images of a person wearing a transparent mask were presented concurrently with the instruction text for participants who were unfamiliar with transparent mask products. Participants assigned to the no-mask group received no additional instructions such as those presented for the surgical and transparent mask groups. The tasks for each trial were the same as those for the practice trials: emotion categorization and emotion intensity rating tasks (as for an attention check trial, see below). Unlike in the practice trials, a face stimulus was presented with a surgical mask, a transparent mask, or without a face mask, depending on the face mask group. After completing the test trials, participants answered their age (*20–59 years*, *do not want to answer*) and sex (*female*, *male*, *other sex*, *do not want to answer*).

An instructional manipulation check (Oppenheimer et al., 2009) and directed question scale (Maniaci & Rogge, 2014) were administered to check participants’ attention and compliance with the instructions. The instructional manipulation check, which was created in line with a previous study (Miura & Kobayashi, 2016), was inserted after informed consent (i.e., before starting the practice trials). Directed question scales were inserted into the test trials. A neutral face stimulus was presented for the attention check trial. The stimulus was the same as those used in the practice trials and was presented with a surgical mask, a transparent mask, or without a face mask, based on the face mask group. Participants were instructed to respond “surprise” and to adjust the slider's position to “0,” regardless of the actual facial expression and perceived intensity. Participants who failed to pass the attention checks were led to an exit screen, and their data were not collected. Prior to beginning the experiment, participants were informed that the experiment would engage attention checks and that the experiment would be terminated midway if they failed to pass the attention checks, in which case they would not receive payment.

#### Data Exclusion

Five hundred and seventy-nine participants accessed the platform for the experiment. However, the data from 318 participants (see *n* below for breakdown) were removed based on the acceptance criteria. Before starting the experiment, the following four criteria were determined: (a) participants who failed to pass the attention checks (*n* = 280); (b) participants with extremely low accuracy or low intensity (<2 *SD* from the group mean) in emotion categorization or emotion intensity rating tasks in seven practice trials (*n* = 28); (c) participants with extremely low accuracy or low intensity (<2 *SD* from the group mean) in emotion-categorization or emotion intensity rating tasks in 28 test trials (no-mask group: *n* = 4, surgical mask group: *n* = 1, transparent mask group: *n* = 5), and (d) participants who recorded systematic responses (returning the same value, for example, all 0s, 50s, or 100s; none of the participants returned such systematic responses). The criteria (b) and (c), based on the traditional standard (i.e., <2 *SD* from the mean), were adopted as it was expected that some participants would possess an extremely low ability to read emotions, or would answer carelessly or sloppily even after passing the attention checks.

### Results

#### Emotion Recognition

**Figure 2.**
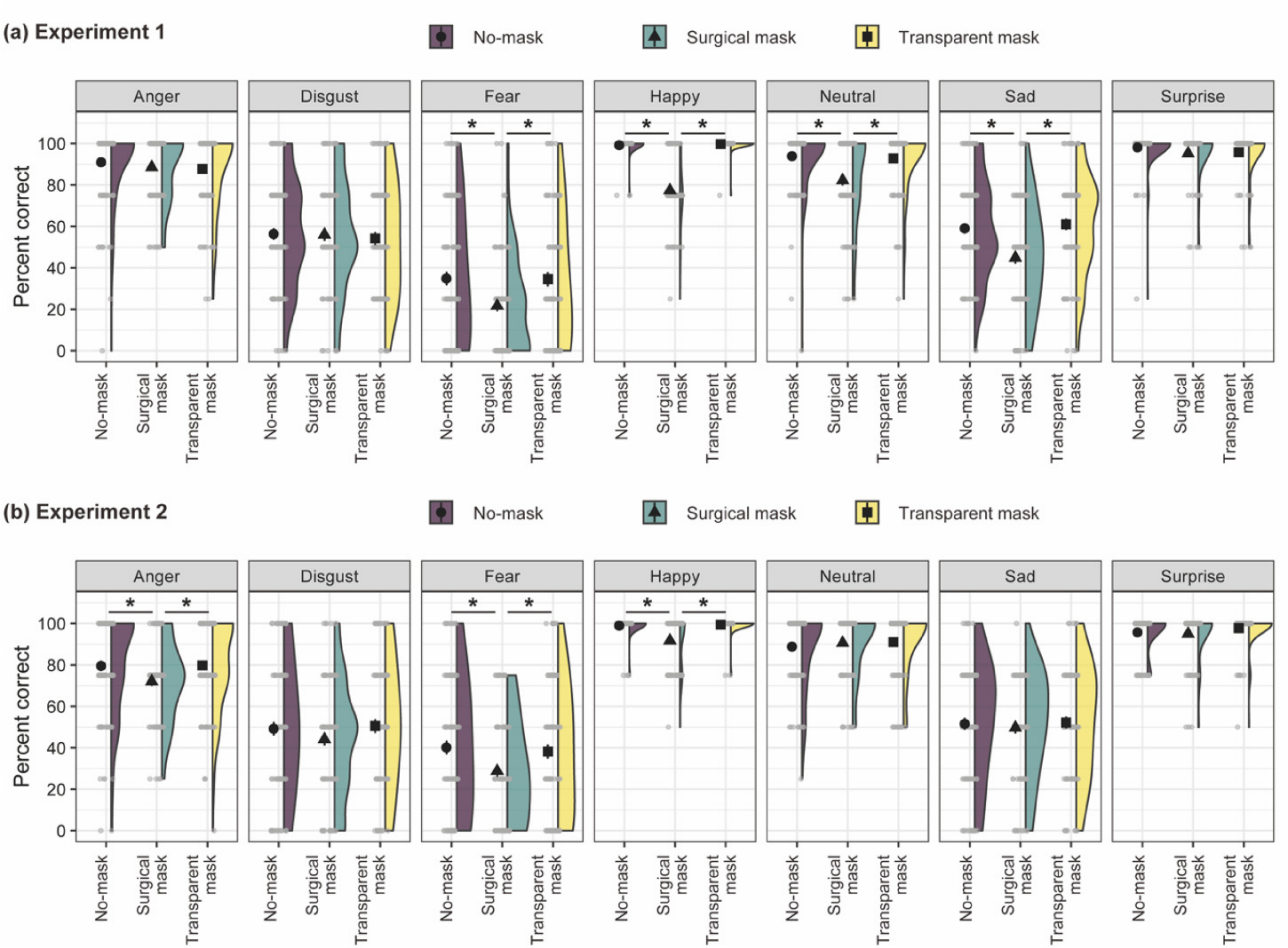
Means of percent corrects for the emotion categorization as a function of the face mask and the facial expression in Experiments 1 and 2 are shown in the top (a) and bottom (b), respectively. Black points with bars represent means ± 1 standard error. Gray dots depict single data points. Half violin plots depict kernel densities of data. *p < .05.

All analyses were conducted using the HAD (Version 17; Shimizu, 2016). First, we conducted a two-way mixed ANOVA for the accuracy of the emotion categorization task (Figure 2(a)). The ANOVA showed a main effect of face mask, *F*(2, 258) = 32.08, *p* < .001, η_p_^2^ = .20, and facial expression, *F*(6, 1548) = 383.71, *p* < .001, η_p_^2^ = .60. A significant interaction was also identified, *F*(12, 1548) = 4.85, *p* < .001, η_p_^2^ = .04.

To identify the nature of the main effect of facial expression, a simple effect of a face mask on each facial expression was tested. The effects of a face mask differed across facial expressions: significant effects were observed for fear, *F*(2, 258) = 5.49, *p* = .005, η_p_^2^ = .04, happy, *F*(2, 258) = 152.79, *p* < .001, η_p_^2^ = .54, neutral, *F*(2, 258) = 10.67, *p* < .001, η_p_^2^ = .08, and sad faces, *F*(2, 258) = 10.54, *p* < .001, η_p_^2^ = .08, but not for anger, disgust, and surprise faces, *F*s(2, 258) < 1.63, *p*s > .199, η_p_^2^s < .02.

Multiple comparisons (modified Shaffer's method) revealed that accuracy was lower in the surgical mask group than in the no-mask group (fear face, *t*(258) = 2.96, *p* = .003, *g* = 0.44; happy face, *t*(258) = 15.26, *p* < .001, *g* = 2.29; neutral face, *t*(258) = 4.24, *p* < .001, *g* = 0.64; sad face, *t*(258) = 3.78, *p* < .001, *g* = 0.57). Notably, the impairments disappeared while wearing a transparent mask: accuracy was higher in the transparent mask group than surgical mask group (fear face, *t*(258) = 2.81, *p* = .005, *g* = 0.43; happy face, *t*(258) = 15.19, *p* < .001, *g* = 2.34; neutral face, *t*(258) = 3.76, *p* < .001, *g* = 0.58; sad face, *t*(258) = 4.19, *p* < .001, *g* = 0.65) and the significant differences were not observed between the no-mask and transparent mask groups (fear face, *t*(258) = 0.07, *p* = .943, *g* = 0.01; happy face, *t*(258) = 0.35, *p* = .726, *g* = 0.05; neutral face, *t*(258) = 0.37, *p* = .709, *g* = 0.06; sad face, *t*(258) = 0.53, *p* = .599, *g* = 0.08).

#### Emotion Intensity

**Figure 3.**
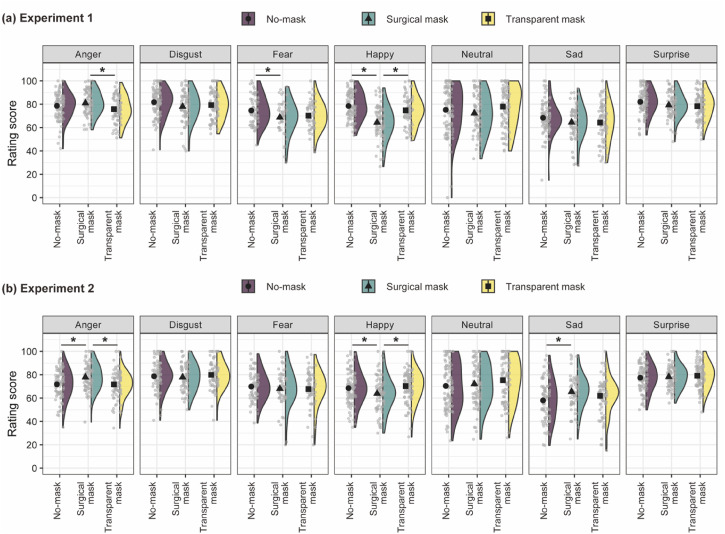
Means of rating scores for the perceived intensity of emotions as a function of the face mask and the facial expression in Experiments 1 and 2 are shown in the top (a) and bottom (b), respectively. Black points with bars represent means ± 1 standard error. Gray dots depict single data points. Half violin plots depict kernel densities of data. *p < .05.

A two-way mixed ANOVA was conducted for the rating scores of the perceived intensity of emotion (Figure 3(a)). Rating scores for each facial expression were averaged for correct trials of the emotion categorization task. For comparison among the mask groups, for instance, it would not be equivalent to compare the rating scores where a fear face was correctly categorized with those where a fear face was miscategorized as a surprise face (e.g., as a result of wearing a surgical mask). Due to this, the rating scores of correct trials were only used. Because there were missing values for some facial expressions (i.e., recognition accuracy of some participants in the categorization task was 0% for a specific facial expression), data from 148 participants out of 261 participants ran in the ANOVA after treatment with list-wise deletion. A significant main effect of facial expression, *F*(6, 870) = 36.61, *p* < .001, η_p_^2^ = .20, and a significant interaction, *F*(12, 870) = 4.07, *p* < .001, η_p_^2^ = .05, were identified, although the main effect of face mask was not significant, *F*(2, 145) = 1.13, *p* = .326, η_p_^2^ = .02.

A simple effect of a face mask was tested for each facial expression. Because the number of missing values varied with facial expressions, the number of data used in the tests varied according to the facial expressions. A significant effect was identified for the anger, *F*(2, 257) = 4.83, *p* = .009, η_p_^2^ = .04, *n* = 260, fear, *F*(2, 165) = 3.32, *p* = .039, η_p_^2^ = .04, *n* = 168, and happy faces, *F*(2, 258) = 28.78, *p* < .001, η_p_^2^ = .18, *n* = 261. The significant effects were not found in the disgust, *F*(2, 244) = 2.25, *p* = .108, η_p_^2^ = .02, *n* = 247, neutral, *F*(2, 257) = 2.05, *p* = .131, η_p_^2^ = .02, *n* = 260, sad, *F*(2, 245) = 2.24, *p* = .108, η_p_^2^ = .02, *n* = 248, and surprise faces, *F*(2, 258) = 2.98, *p* = .052, η_p_^2^ = .02, *n* = 261.

Multiple comparisons revealed that the perceived intensity of emotion for the happy face was lower in the surgical mask group than in the no-mask, *t*(258) = 7.39, *p* < .001, *g* = 1.11, and transparent mask groups, *t*(258) = 5.30, *p* < .001, *g* = 0.82. For the happy face, there was no significant difference between the no-mask and transparent mask groups, *t*(258) = 1.95, *p* = .052, *g* = 0.29. For the anger face, the perceived intensity of emotion in the surgical mask group was higher than that in the transparent mask group, *t*(257) = 3.10, *p* = .002, *g* = 0.48. There were no significant differences between the surgical mask and no-mask groups (*t*(257) = 1.47, *p* = .143, *g* = 0.22., and between the no-mask and transparent mask groups, *t*(257) = 1.72, *p* = .087, *g* = 0.26. For the fear face, the perceived intensity of emotion was lower in the surgical mask group than in the no-mask group, *t*(165) = 2.40, *p* = .018, *g* = 0.46. The difference between the surgical mask and transparent mask groups, *t*(165) = 0.57, *p* = .568, *g* = 0.11, and that between the no-mask and transparent mask groups was not identified, *t*(165) = 1.91, *p* = .057, *g* = 0.35.

### Discussion

We found that wearing a surgical mask and a transparent mask had different effects on reading facial expressions. The accuracy of emotion recognition for fear, happy, neutral, and sad faces was impaired by wearing a surgical mask, compared to not wearing a mask. In addition, the perceived intensities of happy and fear emotions were reduced by wearing a surgical mask. Importantly, these negative effects were not observed when the models wore a transparent mask. The mean values in the transparent mask group were equivalent to those in the no-mask group.

In Experiment 2, we tested whether the results of Experiment 1 could be replicated and generalized using male face stimuli. Although past findings that reading of facial expressions is impaired by wearing a surgical mask were generally replicated in Experiment 1, the results somewhat differed from those of previous studies (Carbon, 2020; Marini et al., 2021; Noyes et al., 2021; Parada-Fernández et al., 2022; Pazhoohi et al., 2021; Ramachandra & Longacre, 2022). For example, Carbon (2020) reported decreased recognition accuracies for anger, disgust, happy, and sad faces with mask-wearing. Due to these inconsistencies with previous studies on the effects of wearing a surgical mask on the perception of facial expressions, we decided to examine the robustness of the surgical and transparent mask-wearing effects in Experiment 2.

## Experiment 2

### Method

Experiment 2 followed preregistration on AsPredicted (https://osf.io/md8g4/). The methods were identical to Experiment 1, except for the participants and face stimuli. Participants were 268 volunteers (188 females, 79 males, and 1 other sex; *M*_age_ = 39.7 years and *SD*_age_ = 9.4 years) who did not participate in Experiment 1. They were randomly assigned to one of the three face mask groups (no-mask group, *n* = 94; surgical mask group, *n* = 85; transparent mask group, *n* = 89). Face stimuli were replaced with four male face images (Y16M, Y24M, Y55M, and Y58M) selected from the Tsinghua facial expression database (Yang et al., 2020) with permission. Another face image (Y57M) was selected for practice trials and an attention check trial. Figure 1(b) shows the face stimuli. They can also be seen on the OSF (https://osf.io/ydtev/).

Although 603 participants accessed the platform for the experiment, the data from 335 participants (see *n* below for breakdown) were removed based on the data acceptance criteria. The criteria were the same as those of Experiment 1: (a) participants who failed to pass the attention checks (*n* = 301); (b) participants with extremely low accuracy or low intensity in the practice trials (*n* = 17); and (c) participants with extremely low accuracy or low intensity in the test trials (no-mask group, *n* = 4; surgical mask group, *n* = 6; transparent mask group, *n* = 7). None of the participants returned systematic responses.

### Results

#### Emotion Recognition

We first conducted a two-way mixed ANOVA for the accuracy of the emotion categorization task (Figure 2(b)). The ANOVA revealed a significant main effect of face mask, *F*(2, 265) = 9.24, *p* < .001, η_p_^2^ = .07, and facial expression, *F*(6, 1590) = 339.84, *p* < .001, η_p_^2^ = .56. However, the interaction was not significant, *F*(12, 1590) = 0.97, *p* = .459, η_p_^2^ = .01.

Based on registration, a simple effect of the face mask on each facial expression was tested, although the interaction was not significant. Significant simple effects were identified for the anger, *F*(2, 265) = 3.24, *p* = .041, η_p_^2^ = .02, fear, *F*(2, 265) = 3.54, *p* = .030, η_p_^2^ = .03, and happy faces, *F*(2, 265) = 25.57, *p* < .001, η_p_^2^ = .16. There were no significant effects for the disgust, neutral, sad, and surprise faces, *F*s(2, 265) < 1.80, *p*s > .168, η_p_^2^s < .02.

Multiple comparisons (modified Shaffer's method) revealed that accuracy was lower in the surgical mask group than in the no-mask group (anger face, *t*(265) = 2.20, *p* = .029, *g* = 0.33; fear face, *t*(265) = 2.51, *p* = .013, *g* = 0.37; happy face, *t*(265) = 6.05, *p* < .001, *g* = 0.90). The impairments were moderated by wearing a transparent mask: accuracy was higher in the transparent mask group than in the surgical mask group (anger face, *t*(265) = 2.24, *p* = .026, *g* = 0.34; fear face, *t*(265) = 2.05, *p* = .041, *g* = 0.31; happy face, *t*(265) = 6.39, *p* < .001, *g* = 0.97). No significant differences were observed between the no-mask and transparent mask groups (anger face, *t*(265) = 0.08, *p* = .940, *g* = 0.01; fear face, *t*(265) = 0.44, *p* = .661, *g* = 0.06; happy face, *t*(265) = 0.43, *p* = .668, *g* = 0.06).

#### Emotion Intensity

Rating scores for perceived intensity for each emotion were calculated using the same procedure followed in Experiment 1. We conducted a two-way mixed ANOVA (*n* = 151) for rating scores (Figure 3(b)) after treatment with list-wise deletion (*n* = 117); a significant main effect of facial expression, *F*(6, 888) = 45.98, *p* < .001, η_p_^2^ = .24, and a significant interaction, *F*(12, 888) = 3.13, *p* = .001, η_p_^2^ = .04, were observed, although the main effect of face mask was not identified, *F*(2, 148) = 0.85, *p* = .429, η_p_^2^ = .01.

A simple effect of the face mask on each facial expression was tested. Because the number of missing values differed with facial expressions, the number of data used in the tests varied depending on the facial expressions. Significant simple effects of the face mask were identified for anger, *F*(2, 262) = 7.54, *p* = .001, η_p_^2^ = .05, *n* = 265, happy, *F*(2, 265) = 4.64, *p* = .010, η_p_^2^ = .03, *n* = 268, and sad faces, *F*(2, 237) = 4.38, *p* = .013, η_p_^2^ = .04, *n* = 240. Significant effects were not found for disgust, *F*(2, 224) = 0.51, *p* = .603, η_p_^2^ < .01, *n* = 227, fear, *F*(2, 188) = 0.42, *p* = .658, η_p_^2^ < .01, *n* = 191, neutral, *F*(2, 265) = 1.59, *p* = .206, η_p_^2^ = .01, *n* = 268, and surprise faces, *F*(2, 265) = 0.58, *p* = .559, η_p_^2^ < .01, *n* = 268.

As in Experiment 1, the perceived intensity of emotion for happy faces was lower in the surgical mask group than in the no-mask, *t*(265) = 2.18, *p* = .030, *g* = 0.32, and transparent mask groups, *t*(265) = 2.95, *p* = .003, *g* = 0.45. There was no significant difference between the no-mask and transparent mask groups, *t*(265) = 0.82, *p* = .411, *g* = 0.12. In addition, the results for the anger face observed in Experiment 1 were partially replicated: perceived intensity of emotion was higher in the surgical mask group than in the no-mask group, *t*(262) = 3.37, *p* = .001, *g* = 0.51, and transparent mask group, *t*(262) = 3.39, *p* = .001, *g* = 0.51. No significant difference was found between the no-mask and transparent mask groups in the anger face, *t*(262) = 0.05, *p* = .957, *g* = 0.01. Furthermore, the perceived intensity of emotion for the sad face was also higher in the surgical mask than in the no-mask group, *t*(237) = 2.96, *p* = .003, *g* = 0.47. There were no significant differences between the no-mask and transparent mask groups, *t*(237) = 1.60, *p* = .111, *g* = 0.25, and between the surgical mask and transparent mask groups, *t*(237) = 1.39, *p* = .167, *g* = 0.22.

### Discussion

In Experiment 2, we replicated the results of Experiment 1, in which wearing a surgical mask negatively affected the reading of facial expressions compared to not wearing a mask. Comparing the results of Experiments 1 and 2, wearing a surgical mask robustly impaired emotion recognition for fear and happy faces. Further, it also robustly reduced the perceived emotion intensity for happy faces. Finally, we replicated the results that these impairments observed when wearing a surgical mask were not observed when using a transparent mask.

## Descriptive Analysis for Misclassification

We assessed whether fear and happy faces were confused with each other when wearing a surgical mask and whether the confusion was alleviated by wearing a transparent mask. The reason for focusing on only fear and happy faces was that robust impairments by mask-wearing occurred in these faces in Experiments 1 and 2 (confusion matrices of expressed and perceived emotions, including anger, disgust, neutral, sad, and surprise faces, are shown in Table 1).

**Table 1.** The confusion matrix of expressed emotions (by models) and perceived emotions (by participants) in Experiments 1 and 2 are shown in top (a) and bottom (b), respectively.

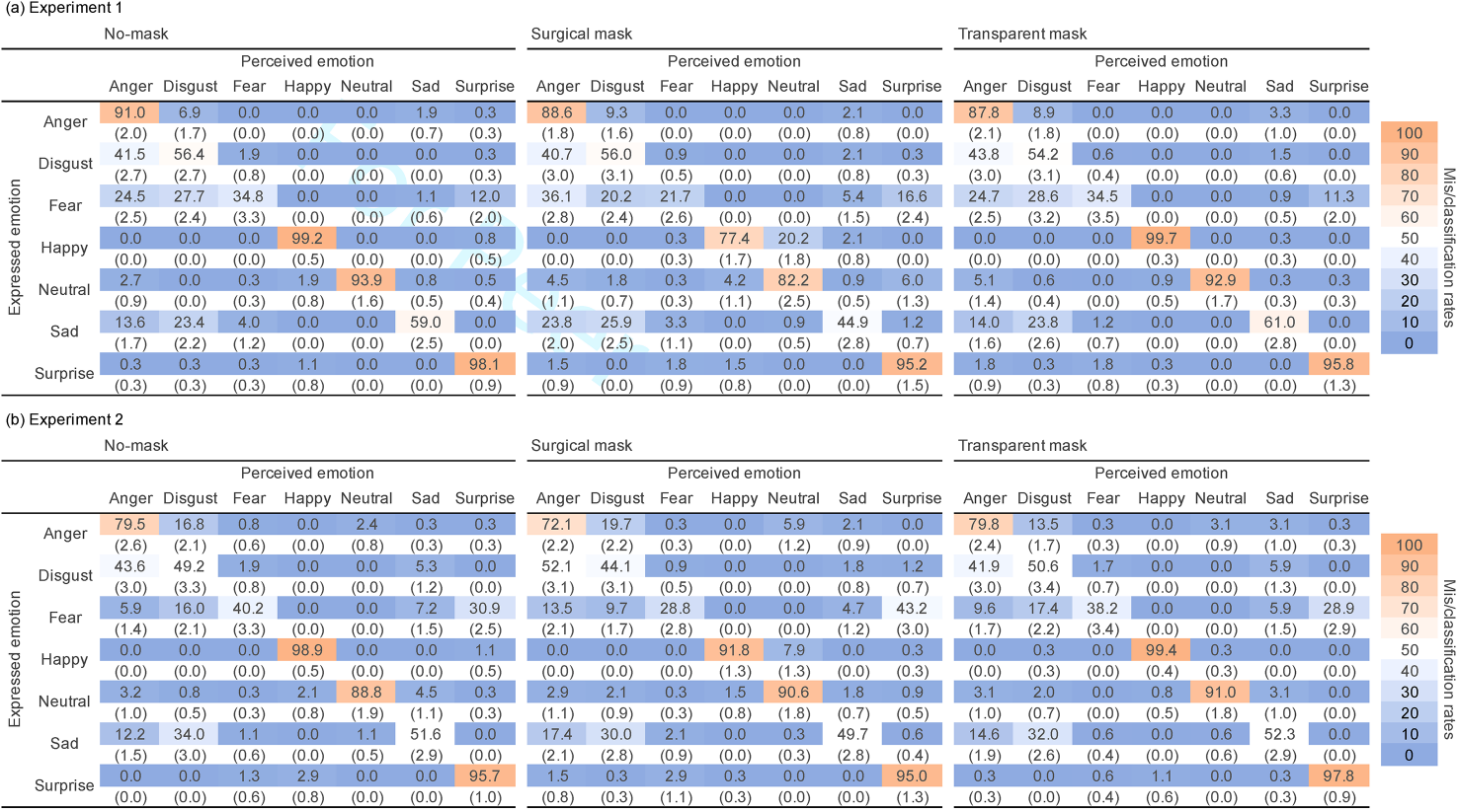

*Note*: The values in the colored cells are means of mis/classification rates (in %) in each emotion. Standard errors are in parenthesis.

Below, we describe two robust misclassifications commonly observed in Experiments 1 and 2. First, happy faces were prone to misclassification as neutral faces when wearing a surgical mask compared to when not wearing a mask. In both Experiments 1 and 2, percent corrects for happy faces (i.e., classification rates for happy faces as happy face) were reduced when wearing a surgical mask (77.4% in Experiment 1; 91.8% in Experiment 2) compared to when not wearing a mask (99.2% in Experiment 1; 98.9% in Experiment 2) or when wearing a transparent mask (99.7% in Experiment 1; 99.4% in Experiment 2). This confusion represented a misclassification of happy faces as neutral. The misclassification rates for happy faces with a surgical mask were 20.2% in Experiment 1 and 7.9% in Experiment 2. In contrast, for those without a mask the misclassification rates were 0% in Experiment 1 and 0% in Experiment 2, and for those with a transparent mask were 0% in Experiment 1 and 0.3% in Experiment 2. Second, fear faces were prone to misclassification as anger or surprise faces. Percent corrects for fear faces were reduced when wearing a surgical mask (21.7% in Experiment 1; 28.8% in Experiment 2) compared to when not wearing a mask (34.8% in Experiment 1; 40.2% in Experiment 2) or when wearing a transparent mask (34.5% in Experiment 1; 38.2% in Experiment 2). The misclassification rates of fear faces as anger faces were higher when wearing a surgical mask (36.1% in Experiment 1; 13.5% in Experiment 2) than when not wearing a mask (24.5% in Experiment 1; 5.9% in Experiment 2) or when wearing a transparent mask (24.7% in Experiment 1; 9.6% in Experiment 2). Similar patterns were observed for the misclassification as surprise faces. The misclassification rates were higher when wearing a surgical mask (16.6% in Experiment 1; 43.2% in Experiment 2) than when not wearing a mask (12.0% in Experiment 1; 30.9% in Experiment 2) or when wearing a transparent mask (11.3% in Experiment 1; 28.9% in Experiment 2).

The misclassification of happy faces as neutral faces was reasonable because the perceived intensity of happy faces decreased in the surgical mask group (Figure 3). The misclassification of fear faces as anger or surprise faces might be due to the perceptual similarity of the visual cues in the upper face regions. Specifically, common action units are involved in the upper face region for these two facial expressions (Ekman & Friesen, 1978), that is, both fear and angry faces recruit the same action units to lower the eyebrows (AU4) and widen the eyes (AU5), and both fear and surprise faces involve the same action units of lifting the eyebrows (AU1 + 2) and widening the eyes (AU5). However, these misclassifications cannot be explained solely by the perceptual similarity, because misclassifications in the opposite direction, such as those of angry or surprise faces as fear faces, were not found in Experiments 1 and 2. We argue that the inaccuracy of emotion recognition for fear faces among East Asian people (Dailey et al., 2010; Jack et al., 2009) might be also involved in the present misclassification of fear faces. Thus, the perceptual similarity between fear and anger or surprise faces, and the ambiguity of recognizing fear faces could have contributed to the present misclassification. Although the interpretation of these misclassifications of fear faces is inconclusive, the most important finding was that these misclassifications were moderated by adopting a transparent mask. The use of a transparent mask instead of a surgical mask could reduce the misclassification of facial expressions.

## Exploratory Analysis

We conducted exploratory analyses to investigate whether the effect of the face mask was modulated by participants’ sex or the face models’ sex, considering the sex differences on emotion recognition ability and emotional expression (Kring & Gordon, 1998; Thompson & Voyer, 2014). In general, these analyses revealed that the impairments in the recognition and perceived intensity of facial emotions in the surgical mask group as compared to those in the no-mask and transparent mask groups were not modulated by participants’ and face models’ sex. We do not discuss the results in more detail because the present study was not designed to examine these effects (see OSF for the details, https://osf.io/4er5v/).

## General Discussion

The present study showed that wearing a surgical mask impaired the performance of reading facial expressions, both with respect to recognition and the perceived intensity of facial emotions. These results are largely consistent with previous studies reporting such impairments due to wearing a surgical mask (Carbon, 2020; Gori et al., 2021; Grenville & Dwyer, 2022; Grundmann et al., 2021; Kim et al., 2022; Marini et al., 2021; Noyes et al., 2021; Parada-Fernández et al., 2022; Pazhoohi et al., 2021; Ramachandra & Longacre, 2022; Sheldon et al., 2021; Tsantani et al., 2022) and demonstrating the importance of the mouth region on emotion recognition (Blais et al., 2012; Bombari et al., 2013; Boucher & Ekman, 1975; Calder et al., 2000; Calvo et al., 2014; Eisenbarth & Alpers, 2011; Hanawalt, 1944; Kotsia et al., 2008; Saumure et al., 2018; Schurgin et al., 2014; Schyns et al., 2002; Wegrzyn et al., 2017). The most important finding of the present study was that these impairments were moderated by wearing a transparent mask. The use of nontransparent face masks during the COVID-19 pandemic made it difficult to read facial expressions. However, such difficulties could be reduced using transparent masks. The replication using face images of East Asian people or recruiting East Asian participants (vs. using face images of Caucasian people and recruitment of Western participants in previous studies) would contribute to the literature examining the effects of wearing masks and mouth occlusion (e.g., Kotsia et al., 2008) on perceived facial expressions.

A recent study investigated the effect of wearing a transparent mask on the recognition of facial emotion among Caucasian face images and Western participants (Marini et al., 2021). They found that wearing a surgical mask (vs. no-mask) impaired recognition accuracy of fear, happy, and sad faces. Impairments were moderated by wearing transparent masks. Although Marini et al.’s study was unavailable when we planned the present study, the most striking difference between the present study and Marini et al.'s study was that we found the effects of wearing a transparent mask not only on recognition of facial emotion, but also on the perceived intensity of emotions. Complementary to Marini et al.'s study, we demonstrated that the reduction in perceived intensity of emotions was also moderated by wearing a transparent mask. The perceived intensity of facial emotions, particularly for happy faces, is critical in judging a person's social aspects (e.g., the person's warmth) in interpersonal situations (Li et al., 2021). Because wearing a surgical mask reduces perceived intensity in both genuine (Duchenne) and social (non-Duchenne) smiles (Sheldon et al., 2021), genuine smiles could be misclassified as social smiles, and social smiles could be viewed as neutral expressions when wearing a surgical mask. Misclassification may negatively affect interpersonal impressions since individuals with genuine smiles are evaluated more positively than those with social smiles (Gunnery & Ruben, 2016). The use of a transparent mask can alleviate such modulations.

The effects of wearing a surgical mask on the recognition and perceived intensity of facial emotions differed according to facial expression. Regarding emotion recognition analyses, we robustly observed that wearing a surgical mask, compared to not wearing a mask, impaired the accuracy of fear and happy faces. These results are consistent with previous findings that recognition of happy (Carbon, 2020; Grenville & Dwyer, 2022; Kim et al., 2022; Marini et al., 2021; Noyes et al., 2021; Parada-Fernández et al., 2022; Pazhoohi et al., 2021; Ramachandra & Longacre, 2022) and fear faces (Marini et al., 2021; Noyes et al., 2021; Pazhoohi et al., 2021; Ramachandra & Longacre, 2022) is affected by mask-wearing. This is compatible with the finding that the mouth region is important for the detection of happy and fear faces (Bombari et al., 2013). With respect to emotion intensity analyses, we robustly observed that the perceived intensity of happy faces was reduced by wearing a surgical mask. These results are consistent with previous findings (Pazhoohi et al., 2021; Ramachandra & Longacre, 2022; Sheldon et al., 2021; Tsantani et al., 2022). Taken together, the inhibition of reading facial expressions by surgical masks was significant and robust for happy faces with regard to both recognition and perceived intensity of facial emotions.

Some results of the current study are inconsistent with those of previous studies. Specifically, we did not find robust results regarding the impairment of emotion recognition of anger, disgust, neutral, sad, and surprise faces (Figure 2), while other researchers reported impairments in recognition of these faces (Carbon, 2020; Grenville & Dwyer, 2022; Kim et al., 2022; Marini et al., 2021; Noyes et al., 2021; Parada-Fernández et al., 2022; Pazhoohi et al., 2021; Ramachandra & Longacre, 2022). For example, Carbon (2020) demonstrated an impairment in recognizing happy, anger, disgust, and sad faces. This inconsistency was also observed for perceived intensity of emotion (Pazhoohi et al., 2021; Ramachandra & Longacre, 2022; Tsantani et al., 2022). In addition, the results are also partially inconsistent with those of a previous study that tested emotion recognition by using the parts of faces (Kotsia et al., 2008), suggesting that the occlusion of mouth impairs the recognition of anger, fear, happy, and sad emotions.

There are two possible explanations for these differences between the present and the previous studies. The first explanation is the existence of cultural differences. East Asians focus more on the eyes than on the mouth when reading emotions, while Westerners see the whole face, including the mouth (e.g., Jack et al., 2009). Since in the present study, the eyes region, as an effective cue for East Asians, is not occluded by a face mask, recognizing emotions on those who wear masks may not be impaired in East Asians compared to Westerners. Due to the difference in the focus of attention, the impairments of emotion recognition might be observed only for fear and happy faces in the present study through recruiting East Asian participants. In a recently published study recruiting South Korean (i.e., East Asian) participants, however, the impairments were found for anger, disgust, happy, sad, and surprise faces (Kim et al., 2022). Therefore, the difference in the results between our and previous studies may not be explained solely by cultural differences. The second explanation relies on study designs. With the exception of one study (Marini et al., 2021), the previous studies employed a within-participants design, while our study employed a between-participants design to manipulate the mask factor. Given that participants saw both faces with a mask and without a mask in the within-participants design, this manipulation provides participants with a clue as to what the study was trying to assess. In previous studies, demand characteristics highlighted the difference between mask-wearing and no-mask conditions.

Interestingly, the perceived intensity of emotion for anger faces tended to be enhanced when wearing a surgical mask compared to a transparent mask or no-mask (Figure 3, left-most plot). This enhancement was significant for male faces (Experiment 2). For female faces (Experiment 1), mean scores indicated a similar trend, although the difference between conditions did not reach the significance level. Taken together, these results suggest that wearing a surgical mask may increase the perceived intensity of negative facial expressions. Ramachandra and Longacre (2022) reported such an increase in perceived intensity of emotion for fear faces, not anger faces (strictly speaking, they did not use mask-wearing face images but cropped images of the lower region of the face, i.e., images of eyes only). In Experiment 1 of the present study, however, we found the opposite results: wearing a surgical mask reduced the perceived intensity of fear faces (although these were not replicated in Experiment 2). Future efforts should be directed to examine whether the inconsistencies between the studies are due to cultural differences (e.g., East Asians are not as good at recognizing fear faces as Westerners, (Dailey et al., 2010; Jack et al., 2009) or differences in the face databases used.

Two limitations remain due to the present manipulation that the mask-wearing faces underwent through graphic editing. The first limitation is the stimulus representativeness of the transparent mask-wearing faces. When transparent masks are used in real life, the plastic surface reflects light in different ways depending on the lighting conditions. Such reflections might then alter facial appearance via the transparent mask. Nonetheless, the mouth region is clearly visible when the present transparent mask is used in real life. Actual examples of the use of transparent masks are available in the Supplemental Material (see OSF, https://osf.io/r2dvb/). However, it is true that the face stimuli used in the present study consisted of snapshots taken under good lighting conditions. Thus, it is unknown whether the results of the present study can be generalized to any transparent mask-wearing faces captured under a variety of lighting environments. Another limitation is the possibility that people communicate differently when they are wearing a face mask compared to when they are not wearing it. Specifically, expressions around the eye region differ with and without a face mask during communication (Okazaki et al., 2021). In the current study, the images of masked faces were digitally created by superimposing a surgical mask image on images of bare faces. Thus, it should be acknowledged that such digitally processed mask-wearing faces can be different from mask-wearing faces captured spontaneously during communication (see also Grenville & Dwyer, 2022). To address these two limitations, additional studies comparing differences in emotion recognition with and without a face mask under actual communication situations as well as under different lighting conditions (or using video stimuli recording these) would be needed.

The use of nontransparent masks contributed to the problem of occluding facial expressions during the COVID-19 pandemic. Using transparent masks could solve the problem. Being able to see facial expressions forms a social bond that connects people. Lack of face-to-face contact, in turn, influences psychological disengagement and distress (e.g., PPE portrait project, 2021). Previous studies about the occlusion of facial expressions have been practiced in introducing a face shield or attaching a face portrait to the chest (Molnar-Szakacs et al., 2021; Reidy et al., 2020; Wiesmann et al., 2021). However, the use of a face shield alone is not effective in protection against COVID-19 (Verma et al., 2020). The use of face portraits does not allow the transmission of dynamic facial information that is important to convey facial emotions (Ambadar et al., 2005; Edwards, 1998; Kamachi et al., 2001). Because transparent masks make the entire face visible, provide protection, and are able to convey dynamic facial information, they can be used in a range of situations where face-to-face communication is important during the COVID-19 pandemic, including in the contexts of medical care, elderly welfare, education, childcare, and communication with a hearing-impaired person.
